# Viral aetiology of severe acute respiratory illness among patients admitted during the 2022 peri-Hajj period

**DOI:** 10.1016/j.ijregi.2023.05.004

**Published:** 2023-05-21

**Authors:** Abdullah M. Assiri, Haleemah Alsuraihi, Amal Mohammad Mubark Alshahrani, Saleh Zaid Alzaid, Ahmed Mohammed Albarraq, Sari Asiri, Abdullah Rshoud Algwizani, Adel Alotaibi, Jaffar A. Al-Tawfiq

**Affiliations:** a Saudi Ministry of Health, Riyadh, Saudi Arabia; b Public Health Authority, Riyadh, Saudi Arabia; c Infectious Disease Unit, Specialty Internal Medicine, and Quality and Patient Safety Department, Johns Hopkins Aramco Healthcare, Dhahran, Saudi Arabia; d Division of Infectious Diseases, Indiana University School of Medicine, Indianapolis, IN, USA; e Division of Infectious Diseases, Johns Hopkins University, Baltimore, MD, USA

## Abstract

**Introduction:**

Severe acute respiratory illness (SARI) among pilgrims continues to be an important healthcare issue. The aim of this study was to describe the viral aetiology of patients admitted to hospitals in the holy cities of Makkah and Madinah during the 2022 peri-Hajj period.

**Methods:**

This is a retrospective analysis of patients admitted to hospitals with SARI. Patients were tested with multiplex polymerase chain reaction for the most common viral aetiologies.

**Results:**

In total, 179 cases of SARI were identified during the study period. Of these, 101 (56.4%) were males, 78 (43.6%) were females, and 78 (43.6%) were Saudi. The mean age was 58.60 years (standard deviation 20.5) years. The most common age group was ≥65 years (*n*=68, 36%), followed by 55–59 years (*n*=37, 19%). The most common comorbidities were diabetes mellitus (*n*=67, 36%), hypertension (*n*=65, 35%) and chronic lung disease (*n*=34, 18%). Eighty-five (47.5%) patients tested negative and 94 (42.5%) tested positive for various viral aetiologies. The most frequently detected viruses were severe acute respiratory syndrome coronavirus-2 (SARS-CoV-2) (*n*=28, 15%) and influenza (*n*=22, 12%); of the influenza cases, 16 were influenza A (6 (43%) were H3N2), and six were influenza B. The only case of Middle East respiratory syndrome coronavirus (MERS-CoV) was in a citizen, and none of the visitors or residents had MERS-CoV. Of the total cases, 27 (14%) died during the follow-up period. In a binary regression analysis, only age was associated with mortality (*P*=0.002).

**Conclusion:**

The most commonly detected viruses among patients admitted to hospital with SARI were SARS-CoV-2 and influenza. It is important to continue surveillance of admitted and non-admitted patients in different Hajj periods to identify any shift in the aetiologic agents.

## Highlights

• 179 cases of severe acute respiratory illness were included in this study.
• The most common viruses were severe acute respiratory syndrome coronavirus-2 (15%) and influenza (12%).
• 14% of participants died during the follow-up period.
• Only age was associated with mortality (*P*=0.002).

## Introduction

Mass gatherings pose a health risk to the host country as well as other countries upon the return of travellers to their respective countries [1]. The risk of the occurrence and spread of respiratory diseases at mass gatherings has been studied previously, including specific studies related to H1N1 influenza, Middle East respiratory syndrome coronavirus (MERS-CoV) and severe acute respiratory syndrome coronavirus-2 (SARS-CoV-2) [2,3]. The occurrence of acute respiratory tract infections among pilgrims is very common, especially those related to influenza [3]. The annual Hajj pilgrimage is the most studied mass gathering. A systematic review of tested symptomatic pilgrims by polymerase chain reaction (PCR) found that the most common viruses were rhinovirus (5.9–48.8%), influenza virus (4.5–13.9%) and non-MERS coronaviruses (2.7–13.2%) [4].

The coronavirus disease 2019 (COVID-19) pandemic reshaped the world and resulted in the cancellation and downscaling of many mass gatherings around the globe. This was also true for the annual Hajj pilgrimage, which takes place each year in the Kingdom of Saudi Arabia. The emergence of SARS-CoV-2 resulted in a limited number of pilgrims attending the 2020 Hajj, with gradual scaling up of the Hajj in 2021 and 2022 [2]. One study of returning travellers in 2022 showed that 6.7% tested positive for SARS-CoV-2, 0.7% tested positive for influenza virus, and 0.2% tested positive for both SARS-CoV-2 and influenza [5]. Although the aetiology of symptomatic pilgrims has been studied extensively, the aetiology of patients admitted to hospital has received less attention. A previous retrospective study of pilgrims admitted to hospital between 2004 and 2013 only examined bacterial aetiology of pneumonia [6], and found that the most common bacterial aetiologies were *Klebsiella pneumoniae, Streptococcus pneumoniae, Haemophilus influenzae, Staphylococcus aureus* and *Pseudomonas aeruginosa* [6]*.* Another study during the MERS-CoV period showed that the most common viruses among 38 pilgrims with severe pneumonia were human rhinovirus (57.7%), influenza A virus (23.1%) and human coronaviruses (19.2%) [7]. As such, this study was undertaken to evaluate the occurrence and outcome of patients admitted to hospitals during the 2022 peri-Hajj period.

## Methods

Patients diagnosed with severe acute respiratory illness (SARI) who were admitted to the Hajj hospitals during the 2022 peri-Hajj period (14 June 2022 to 8 August 2022) were included in this study. These patients were citizens and residents of the two holy cities, and Hajj pilgrims. Patients who underwent viral testing but did not require hospital admission, and patients who did not meet the definition of SARI were excluded from this study. Nasopharyngeal secretion (NPS) samples, collected as part of routine care, were tested for detection and differentiation of the most common viral and bacterial respiratory organisms.

NPS swabs were collected in transport medium intended for standard-of-care testing for SARS-CoV-2 and other viruses. Specimens were kept at room temperature for ≤4 h, or kept at 4°C for ≤3 days. Frozen specimens were shipped in batches to the public health laboratory on dry ice, and transferred directly to a freezer (<−70°C) until testing.

BioFire Respiratory Panel 2.1 plus was used for the detection of respiratory viruses. The panel is a CE-marked multiplexed nucleic acid test intended for simultaneous qualitative detection and differentiation of nucleic acids for 22 of the most common viral and bacterial respiratory organisms, including SARS-CoV-2, MERS-CoV, adenovirus, coronavirus 229E, coronavirus HKU1, coronavirus NL63, coronavirus OC43, human metapneumovirus, human rhinovirus/enterovirus, influenza A (H1, H3 and H1-2009), influenza B, parainfluenza virus 1–4, respiratory syncytial virus, *Bordetella parapertussis, Bordetella pertussis, Chlamydia pneumoniae* and *Mycoplasma pneumoniae.* NPS swabs were obtained from individuals by their healthcare provider. Approximately 300 μL of each specimen received in the laboratory was subject to testing. The test consists of automated nucleic acid extraction, reverse transcription, nucleic acid amplification and automated results analysis, and takes approximately 45 min per run (i.e. per specimen). If either internal control fails, the software automatically provides a result of ‘invalid’ for all panel analytes. Analytes are reported qualitatively as ‘detected’, ‘not detected’ or ‘equivocal’ (only applicable to influenza A). The test contains two independent assays for the identification of SARS-CoV-2 (one assay targeting the M gene and one assay targeting the S gene). A positive result for either assay provides a detected result for SARS-CoV-2. Demographic and limited clinical data were collected, including date of specimen collection, age, gender and outcome. The study was approved by the institutional review board of the Ministry of Health (IRB 23-15 E).

## Results

In total, 179 cases of admitted patients with SARI were identified during the study period. Of the cases, 101 (56.4%) were males, 78 (43.6%) were females, and 78 (43.6%) were Saudi. The mean age was 58.6 (standard deviation 20.5) years. The most common age group was ≥65 years (*n*=68, 36%), followed by 55–59 years (*n*=37, 19%). The most common comorbidities were diabetes mellitus (*n*=67, 36%), hypertension (*n*=65, 35%) and chronic lung disease (*n*=34, 18%). Of the participants, there were 78 citizens, 40 residents and 61 visitors.

**Figure 1:**
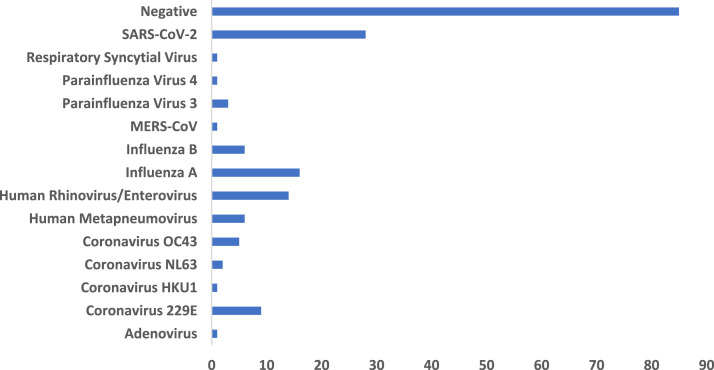
Result of viral polymerase chain reaction test among patients admitted to hospital with severe acute respiratory illness, showing numbers in each category. SARS-CoV-2, severe acute respiratory syndrome coronavirus-2; MERS-CoV, Middle East respiratory syndrome coronavirus.

Eighty-five (47.5%) participants tested negative and 94 (42.5%) tested positive for various viral aetiologies (Figure 1). The most commonly detected viruses were SARS-CoV-2 (*n*=28, 15%) and influenza (*n*=22, 12%); of the influenza cases, 14 were influenza A (of which 6 (43%) were H3N2), and six were influenza B. Among the different groups, the most common pathogens were SARS-CoV-2 among citizens (25.6%), and influenza virus among residents and visitors (12.5% and 21.3%, respectively. The only case of MERS-CoV was in a citizen, and none of the visitors or residents tested positive for MERS-CoV. Twenty-seven (14%) of all cases died during the follow-up period. In a binary regression analysis, only age was associated with mortality (*P*=0.002).

## Discussion

During the COVID-19 pandemic, the annual pilgrimage to Makkah (Hajj) continued, but with a limited number of pilgrims and strict precautionary measures. In the 2022 Hajj, Saudi Arabia hosted nearly 1 million pilgrims, representing the most diverse and one of the largest mass gatherings that took place during the COVID-19 pandemic. The pilgrimage was limited to people <65 years of age who had received COVID-19 vaccinations. Historically, pneumonia accounts for 20% of hospital admissions in Hajj [8]. This study, in addition to Hajj pilgrims, included people living inside the Hajj premises, who were admitted with SARI during the 2022 peri-Hajj period. Similar to Hajj pilgrims, those individuals were required to be up to date with vaccinations against COVID-19 and influenza, and may have the same risk during mass gatherings [1].

In this study, 179 patients were admitted to hospital during the 2022 peri-Hajj period. Of these patients, 85 (45.2%) had a negative viral multiplex PCR test. The most commonly detected viruses were SARS-CoV-2 (15%) and influenza (12%); of the influenza cases, 16 were influenza A, of which 43% were H3N2, and six were influenza B. And none of the admitted pilgrims with SARI had MERS-CoV, thus, the study confrims previous studies of abscence of MERS-CoV among pilgrims. In a previous study in 2022, 76 (7.6%) of 1003 returning pilgrims tested positive for respiratory viruses, as follows: 67 (6.7%) SARS-CoV-2, seven (0.7%) influenza virus, and two (0.2%) both SARS-CoV-2 and influenza virus [5]. The most common variant of SARS-CoV-2 was Omicron sublineage BA.2 [5]. However, these studies differed in design, with patients admitted to hospital in the current study and returning pilgrims in the other study.

The Hajj pilgrimage is attended by millions of people annually, and there is a great deal of physical contact; as such, keeping physical distance is challenging. The 2020 Hajj, which coincided with the emergence of SARS-CoV-2, was significantly limited in numbers [2,9,10], with subsequent escalation following introduction of the COVID-19 vaccine. The case fatality rate in this study was 14%. The case fatality rate is dependent on the aetiologic agent, as well as age and presence of comorbidities.

This study has a few limitations. The study evaluated viral aetiology but did not evaluate all bacterial pathogens among the participants. The test used detects 22 viral and bacterial pathogens, but patients who tested negative may have been infected/colonized with other pathogens that were not detected with this test. Other pathogens, such as *Mycobacterium tuberculosis* and fungi, have been reported in previous studies. In addition, this study only included SARI patients who were admitted to hospitals; patients who were evaluated and discharged were not included. The number of patients evaluated and discharged usually exceeds the number of patients admitted to hospital, and the former group have a different spectrum of pathogens, as shown in other studies [4]. As respiratory tract illness among pilgrims is common, it is prudent to continue surveillance, implementation of strategies for the prevention of such illnesses, and the impact of vaccination and other preventative measures.

## Conflict of interest statement

None declared.
